# Supplementary material for: Novel C1q receptor-mediated signaling controls neural stem cell behavior and neurorepair
Source: eLife. 2020 Sep 7;9:e55732. doi: 10.7554/eLife.55732 (PMC7476762; doi:10.7554/eLife.55732)
Supplement: Supplementary file 1. [file elife-55732-supp1.docx]

| **Supplementary File 1. Number of animals per group for in vivo studies** | | | | | | |
| --- | --- | --- | --- | --- | --- | --- |
| **Experiment Description** | **Group** | **Time of Sacrifice** | ***N*** | **Tissue sectioning/purpose** | **Behavior** | **Exclusions** |
| **C1q expression in the injury epicenter.** | Contusion injury | 3h, 24h, 9d post injury | *12* | 3mm sections. Total protein extraction | None | None |
|  | Laminectomy only (sham) | 3h, 24h, 9d post injury | *4* | 3mm sections. Total protein extraction | None | None |
| **CD44 WT and KO hNSC transplantation into the acute SCI microenvironment** | CD44 WT hNSCs | 16wk post-transplant | *10* | Transverse fate and lesion quantification *(N=5-6)* behavior *(N=10)* | BMS, Ladder Beam. | N = 1 excluded. 2SDs outside the group mean. |
|  | CD44 KO hNSCs | 16wk post-transplant | *8** | Transverse fate and lesion quantification *(N=5-6)*  behavior *(N=8)** | BMS, Ladder Beam. | N=1 excluded. 2SDs outside the group mean  *N=2 mice exhibited  progressive decline  in activity and were excluded and sacrificed. |
|  | Vehicle | 16wk post-transplant | *10* | Transverse fate and lesion quantification *(N=6),* behavior *(N=10)* | BMS, Ladder Beam. | None |
| **C1q-epicenter blockade in acute SCI microenvironment** | α-C1q Ab + hNSCs | 12wk post-transplant | 6 | Transverse fate and lesion quantification, behavior | BMS, Catwalk | None |
|  | Vehicle + hNSCs | 12wk post-transplant | *5* | Transverse fate and lesion quantification, behavior | BMS, Catwalk* | None  *N=2 mice did not reach sufficient BMS score (5) to be tested in catwalk. |
