# Supplementary material for: Novel C1q receptor-mediated signaling controls neural stem cell behavior and neurorepair
Source: eLife. 2020 Sep 7;9:e55732. doi: 10.7554/eLife.55732 (PMC7476762; doi:10.7554/eLife.55732)
Supplement: Supplementary file 2. [file elife-55732-supp2.docx]

| **Supplementary File 2. List of antibodies and concentrations used for immunodetection** | | | | | | | |
| --- | --- | --- | --- | --- | --- | --- | --- |
| **Experiments** | **Antibody** | **Host** | **Manufacturer** | **Catalog number** | **Dilution** | **Specificity of Primary Abs**  **(Purpose)** | **Secondary Antibody** |
| **Histology**  **in vivo** | STEM121 | Mouse | Takara | Y40410 | 1:3000 | Human Cytoplasm | Biotinylated donkey anti-mouse Fab fragment (1:500) |
|  | STEM123 | Mouse | Takara | Y40420 | 1:3000 | Human Astrocytes | Biotinylated donkey anti-mouse Fab fragment (1:500) |
|  | Fibronectin | Rabbit | Sigma Aldrich | F3648 | 1:500 | Fibronectin containing lesion | Biotinylated donkey anti-Rabbit Fab fragment (1:500) |
| **Western Blot**  **In vitro and in vivo** | α-C1q | Mouse | Abcam | ab71940 | 1:200 | Human C1q and Mouse C1q in vivo | ECL donkey anti-mouse IgG, HRP-linked whole Ab (1: 5,000) |
|  | α-C1q | Mouse | Abcam | ab71940 | 1:50 | Far Western-blot | ECL donkey anti-mouse IgG, HRP-linked whole Ab (1: 5,000) |
|  | p-ERK | Rabbit | Cell Signaling | 4370 | 1:1000 | Phosphorylated ERK  (intracellular signaling activation) | ECL donkey anti-rabbit IgG, HRP-linked whole Ab (1: 5,000) |
|  | p-Akt | Rabbit | Cell Signaling | 9916 | 1:1000 | Phosphorylated Akt  (intracellular signaling activation) | ECL donkey anti-rabbit IgG, HRP-linked whole Ab (1: 5,000) |
|  | CD44 | Rabbit | Abcam | ab51037 | 1:1000 | CD44 protein expression | ECL donkey anti-rabbit IgG, HRP-linked whole Ab (1: 5,000) |
|  | β-Actin | Mouse | Sigma Aldrich | A1978 | 1:1000 | β-Actin | ECL donkey anti-mouse IgG, HRP-linked whole Ab (1: 10,000) |
| **Immunocytochemistry and proximity ligation assay (PLA)** | CD44  (hNSC) | Rabbit | Abcam | ab51037 | 1:500 | CD44 protein expression and PLA | Alexa Fluor 555-conjugated donkey anti-rabbit (1: 1,000) or Anti Rabbit (-) PLA probe (1:5) (Sigma) |
|  | CD44  (mNSC) | Rabbit | Abcam | ab189524 | 1:500 | CD44 protein expression in mNSC | Alexa Fluor 555-conjugated donkey anti-rabbit (1: 1,000) |
|  | cMET | Rabbit | Abcam | ab51067 | 1:500 | cMET protein expression and PLA | Alexa Fluor 488-conjugated donkey anti-rabbit (1: 1,000) or Anti Rabbit (-) PLA probe (1:5) (Sigma) |
|  | BAI-1 | Rabbit | Abcam | ab135907 | 1:500 | BAI-1 protein expression and PLA | Alexa Fluor 488-conjugated donkey anti-rabbit (1: 1,000) or Anti Rabbit (-) PLA probe (1:5) (Sigma) |
|  | GPR62 | Rabbit | Thermo Fisher | PA5-33745 | 1:500 | GPR62 protein expression and PLA | Alexa Fluor 488-conjugated donkey anti-rabbit (1: 1,000) or Anti Rabbit (-) PLA probe (1:5) (Sigma) |
|  | ADCY5 | Rabbit | Abcam | ab66037 | 1:500 | ADCY5 protein expression and PLA | Alexa Fluor 488-conjugated donkey anti-rabbit (1: 1,000) or Anti Rabbit (-) PLA probe (1:5) (Sigma) |
|  | α-C1q  (PLA) | Mouse | Abcam | ab71940 | 1:100 | Human C1q and PLA | Anti-Mouse (+) PLA probe (1:5) (Sigma) |
|  | Olig2 | Rabbit | Abcam | ab136253 | 1:1000 | Oligodendrocyte progenitor cells | Alexa Fluor 488-conjugated donkey anti-rabbit (1: 1,000) |
|  | Tubulin βIII | Mouse | Biolegend | MMS-435P | 1:500 | Neurons | Alexa Fluor 488-conjugated donkey anti-mouse (1: 1,000) |
|  | GFAP | Rabbit | Dako | Z 0334 | 1:1000 | GFAP-positive astrocytes | Alexa Fluor 555-conjugated donkey anti-rabbit (1: 1,000) |
| **FLow cytometry,**  **FACS** | CD44-PE | NA | Miltenyi | 130-110-293 | 1:11 | CD44 expression and sorting | N/A |
|  | CD133-FITC | NA | Miltenyi | 130-113-673 | 1:11 | CD133 expression | N/A |
