# Supplementary material for: Novel C1q receptor-mediated signaling controls neural stem cell behavior and neurorepair
Source: eLife. 2020 Sep 7;9:e55732. doi: 10.7554/eLife.55732 (PMC7476762; doi:10.7554/eLife.55732)
Supplement: Supplementary file 3. [file elife-55732-supp3.docx]

| **Supplementary File 3. List of conditions used in vitro for migration, proliferation, fate and signaling assay.** | | | | | |
| --- | --- | --- | --- | --- | --- |
| **Experiment** | **Condition** | **Host species (source)** | **Concentration or dilution used (purpose)** | **Manufacturer** | **Catalog number** |
| **hNSC migration, proliferation, fate and intracellular signaling assays.** | Purified C1q (PMN secreted concentration) | Human | 0.1nM = 28ng/ml | My Biosource | MBS143105 |
|  | Purified C1q (Macrophage/microglia secreted concentration) | Human | 1.0nM = 400ng/ml | My Biosource | MBS143105 |
|  | Purified C1q (C1q plasma concentration) | Human | 200nM = 80µg/ml | My Biosource | MBS143105 |
|  | PD98059 (pERK MAPK inhibitor) | N/A | 10 µM dissolved in DMSO | GIBCO Life Technologies | PHZ1164 |
|  | SB203580 (p38 MAPK inhibitor) | N/A | 10 µM dissolved in DMSO | GIBCO Life Technologies | PHZ1253 |
|  | Pertussis Toxin (GPCR inhibitor) | N/A | 10 µg/ml | GIBCO Life Technologies | PHZ1174 |
|  | DMSO | N/A | 1:1000 | Sigma-Aldrich | D26650 |
| **hNSC migration** | PMN-CM | Mouse | Collected from initial seeding density of 2,500,000 cells/ml* | N/A | N/A |
|  | Mϕ-CM | Mouse | Collected from initial seeding density of 2,500,000 cells/ml* | N/A | N/A |
| **In vivo C1q neutralization** | Anti-C1qNAb | Goat-anti human | 100 µg/ml  (2µl injection) | Quidel | A031 |
