# Supplementary material for: Novel C1q receptor-mediated signaling controls neural stem cell behavior and neurorepair
Source: eLife. 2020 Sep 7;9:e55732. doi: 10.7554/eLife.55732 (PMC7476762; doi:10.7554/eLife.55732)
Supplement: Supplementary file 4. [file elife-55732-supp4.docx]

| **Supplementary File 4. List of primers used in this manuscript** | | | |
| --- | --- | --- | --- |
| **Species** | **Target** | **Forward**  **(sequence) 5’-3’** | **Reverse**  **(sequence) 5’-3’** |
| **Human Primers** | CD44 | CTGCCGCTTTGCAGGTGTA | CATTGTGGGCAAGGTGCTATT |
|  | cMET | AGCGTCAACAGAGGGACCT | GCAGTGAACCTCCGACTGTATG |
|  | BAI-1 | GCGGCGCTACACTCTCTAC | GCACCTCGTCGAAGCTCTC |
|  | GPR62 | ATAGCCGCCTTTCCATCTTGC | CAGCAGCCCGTACAGGAAG |
|  | ADCY5 | GATCGAGGCCATCTCGTTGG | CGTGACATCGTTAGACCAGAC |
|  | GAPDH | AAGGTGAAGGTCGGAGTCAAC | GGGGTCATTGATGGCAACAATA |
| **Mouse primers** | CD44 | CACCATTGCCTCAACTGTGC | TTGTGGGCTCCTGAGTCTGA |
|  | cMET | ATCTCGGAGCCACAAACTACA | CAGTCCCGACAAGGTAAACAAT |
|  | BAI-1 | GGACCCTCGTCGTTACACG | CTGGTAGGTACGAACTCGGC |
|  | GPR62 | CTCAGCGTCACCGAACTCG | GCGCTAAGTAGAAGGCATCTTG |
|  | ADCY5 | AAGATCCTCGGGGATTGTTACT | CTCCCGGACCAACGAGATG |
|  | GAPDH | TGACCTCAACTACATGGTCTACA | CTTCCCATTCTCGGCCTTG |
|  | 18S | AGGGGAGAGCGGGTAAGAGA | GGACAGGATAGGCGGAACA |
