# Supplementary material for: Novel C1q receptor-mediated signaling controls neural stem cell behavior and neurorepair
Source: eLife. 2020 Sep 7;9:e55732. doi: 10.7554/eLife.55732 (PMC7476762; doi:10.7554/eLife.55732)
Supplement: Transparent reporting form [file elife-55732-transrepform.docx]

***eLife’s* transparent reporting form**

We encourage authors to provide detailed information *within their submission* to facilitate the interpretation and replication of experiments. Authors can upload supporting documentation to indicate the use of appropriate reporting guidelines for health-related research (see [EQUATOR Network](http://www.equator-network.org/%20)), life science research (see the [BioSharing Information Resource](https://biosharing.org/)), or the [ARRIVE guidelines](http://www.plosbiology.org/article/info:doi/10.1371/journal.pbio.1000412) for reporting work involving animal research. Where applicable, authors should refer to any relevant reporting standards documents in this form.

If you have any questions, please consult our Journal Policies and/or contact us: [editorial@elifesciences.org](mailto:editorial@elifesciences.org).

**Sample-size estimation**

- You should state whether an appropriate sample size was computed when the study was being designed
- You should state the statistical method of sample size computation and any required assumptions
- If no explicit power analysis was used, you should describe how you decided what sample (replicate) size (number) to use

Please outline where this information can be found within the submission (e.g., sections or figure legends), or explain why this information doesn’t apply to your submission:

For animal studies, animal groups numbers were estimated in base of historical post-hoc power analysis of transplanted hNSC with stereological analyses (Hooshmand et al., 2009) (Article file Lines 889-891)

For in vitro studies, we used N3-4 Biological replicates consistent with accepted standard for in vitro studies (Article file Lines 1254-1255)

**Replicates**

- You should report how often each experiment was performed
- You should include a definition of biological versus technical replication
- The data obtained should be provided and sufficient information should be provided to indicate the number of independent biological and/or technical replicates
- If you encountered any outliers, you should describe how these were handled
- Criteria for exclusion/inclusion of data should be clearly stated
- High-throughput sequence data should be uploaded before submission, with a private link for reviewers provided (these are available from both GEO and ArrayExpress)

Please outline where this information can be found within the submission (e.g., sections or figure legends), or explain why this information doesn’t apply to your submission:

**Replicates**

In each figure legends the number of biological replicates is stated. Additionally, in the Method section a description of biological or technical replicates is stated in each experiment description, and a description of biological versus technical replicates is stated in the statics section (Lines 1249-1253).

C1q signaling by phosphoarray analysis was performed using biological triplicates with technical duplicates (Lines 934-935).

C1q signaling by western blot analysis was performed using biological triplicates (Line 952)

Proximity Ligation Assays were performed with biological duplicates and technical duplicates. (Lines 1073-1074)

Cell motility assays were performed with biological triplicates (Line 1085)

Cell migration assays were performed with biological triplicates or quadruplicates with technical triplicates (Lines 1099-1100)

Cell fate and proliferation analysis were performed with biological triplicate or quadruplicate with technical duplicates (Line 1118)

In experiments including animal models,

All animals per group/strain and exclusion are detailed in supplementary Table 1.

For C1q protein expression analysis in vivo by western blot N=4 mice/timepoint were used (Lines 1225-1227).

For histological analysis N=6 mice/group was used (Lines 1213-1214)

For behavioral analysis N=10 mice/group was used. (Line 1240)

Animal numbers were defined according to previous publications, and estimated in base of historical post-hoc power analysis of transplanted hNSC with stereological analyses (Hooshmand et al., 2009).

**Outliers**

For experiments including animal models. Animal numbers and exclusions are stated in Supplementary Table 1.

In the Method section under Animal Models pre-hoc exclusion criteria and outlier criteria are stated (Lines 882-886).

**Statistical reporting**

- Statistical analysis methods should be described and justified
- Raw data should be presented in figures whenever informative to do so (typically when N per group is less than 10)
- For each experiment, you should identify the statistical tests used, exact values of N, definitions of center, methods of multiple test correction, and dispersion and precision measures (e.g., mean, median, SD, SEM, confidence intervals; and, for the major substantive results, a measure of effect size (e.g., Pearson's r, Cohen's d)
- Report exact p-values wherever possible alongside the summary statistics and 95% confidence intervals. These should be reported for all key questions and not only when the p-value is less than 0.05.

Please outline where this information can be found within the submission (e.g., sections or figure legends), or explain why this information doesn’t apply to your submission:

**Statistical reporting**

In each figure legends the statistical method of analysis and the number of biological replicates is stated. When possible the exact p-values where included.

Additionally, in the Method section a description of biological or technical replicates is stated in each experiment description.

A description of the statistical analysis is stated in Statistic subsection (Article Lines 1249-1262). In all statistical analyses, significance was defined as p ≤ 0.05.

(For large datasets, or papers with a very large number of statistical tests, you may upload a single table file with tests, Ns, etc., with reference to sections in the manuscript.)

**Group allocation**

- Indicate how samples were allocated into experimental groups (in the case of clinical studies, please specify allocation to treatment method); if randomization was used, please also state if restricted randomization was applied
- Indicate if masking was used during group allocation, data collection and/or data analysis

Please outline where this information can be found within the submission (e.g., sections or figure legends), or explain why this information doesn’t apply to your submission:

**Group allocation.**

“All experiments including animal models (animal care, behavior, analysis) where performed by investigators blinded to study groups (masking) and random group allotment was used. Restricted randomization was applied to maintain equal group numbers”. Animal group numbers were defined according to previous publications, and estimated in base of historical post-hoc power analysis of transplanted hNSC with stereological analyses (Hooshmand et al., 2009).

This information can be found in the methods section under “animal models” lines 881 and 882 of the article file.

**Additional data files (“source data”)**

- We encourage you to upload relevant additional data files, such as numerical data that are represented as a graph in a figure, or as a summary table
- Where provided, these should be in the most useful format, and they can be uploaded as “Source data” files linked to a main figure or table
- Include model definition files including the full list of parameters used
- Include code used for data analysis (e.g., R, MatLab)
- Avoid stating that data files are “available upon request”

Please indicate the figures or tables for which source data files have been provided:
